# Effects of chronic sleep deprivation on glucose homeostasis in rats

**DOI:** 10.1007/s41105-016-0061-y

**Published:** 2016-05-31

**Authors:** Xiaowen Xu, Liang Wang, Yan Zhang, Tianjiao Su, Liying Chen, Yan Zhang, Weifeng Ma, Yuanyuan Xie, Tiantian Wang, Fan Yang, Li He, Wenjiao Wang, Xuemei Fu, Hongxia Hao, Yuanzheng Ma

**Affiliations:** 1 Center of Orthopedics, The 309th Hospital of PLA, No. 17 Heishanhu Road, Haidian District, Beijing, 100091 China; 2 Center for Systems Biomedical Sciences, University of Shanghai for Science and Technology, Shanghai, 200093 China; 3 Center of Health Care, The 309th Hospital of PLA, No. 17 Heishanhu Road, Haidian District, Beijing, 100091 China; 4 Director of Division of Science and Technology, National Institute for Nutrition and Food Safety, Chinese Center for Disease Control and Prevention, Beijing, 100050 China

**Keywords:** Chronic sleep deprivation, Glucose intolerance, Insulin resistance, Body weight

## Abstract

Epidemiological studies have shown that chronic sleep disturbances resulted in metabolic disorders. The purpose of this study was to assess the relationship between chronic sleep deprivation (CSD) and the glucose homeostasis in rats. Twenty-four rats were randomly divided into CSD group and control (CON) group. The CSD rats were intervened by a modified multiple platform method (MMPM) to establish an animal model of chronic sleep disturbances. After 3-month intervention, all rats were subjected to an intraperitoneal glucose tolerance test (IPGTT) and an insulin tolerance test (ITT), and the body weight, aspartate aminotransferase (AST), alanine aminotransferase (ALT), creatinine, lipid profile group, and homeostasis model assessment-IR (HOMA-IR) were measured. Both the CSD and CON groups had an attenuation of weight gain after 3-month intervention. The plasma glucose level of CSD group was higher than that of the CON group during the IPGTT (*P* < 0.01). The CSD rats showed a marked increase in HOMA-IR and ITT compared with the CON group (*P* < 0.01). There were no significant differences of AST, ALT, creatinine, and most lipid parameters between the CSD and CON groups (*P* > 0.05). The CSD has a marked effect on glucose homeostasis, comprising glucose intolerance and insulin resistance.

## Introduction

Lack of sleep, especially frequent or chronic sleep disturbances, has become a worldwide health problem, drawing increasingly attention in the modern society. It has been reported that the average sleep duration has decreased from about 9 h per night in 1910 to about 7.04 h presently, and 58 % of people had sleep problems [1, 2]. For the factors of increases in environmental light, longer work days/longer commuting time, an increase in evening and night work, availability of media, and late nocturnal sleep onsets, more people would suffer from chronic sleep deprivation (CSD) [3, 4].

The CSD, leading to both short sleep duration and poor sleep quality, has important consequences for an individual’s well-being [5–7]. In the case of CSD, either short sleep duration or poor quality sleep could affect the neurobiological regulation of circadian rhythm, increasing much burden on the regulatory systems to maintain allostasis [8]. It was also reported that poor sleep may contribute to the chronic, low-grade inflammation associated with an increased risk for future adverse health outcomes [9, 10]. In addition, some research indicated that the CSD may confer increased risk for suicidal behaviors, including suicidal ideation, suicide attempts, and death by suicide [11]. Finally, large-scale experimental studies have proved that the CSD was linked to food intake [12], body weight and energy expenditure [13], metabolic syndrome [14, 15], cardiovascular disease [16], and glucose homeostasis [17–19].

A great number of epidemiologic clinic studies have shown that the CSD leads to both glucose intolerance and insulin resistance [20–25]. Kim et al. reported that short sleep duration (<7 h) was associated with increased risk of impaired fasting glucose (IFG) compared to adequate sleep duration (7–8 h) in men [23]. Matthews et al. found that reduced sleep duration is associated with increased insulin resistance in adolescence [20]. Because of the difficulty to control diet and sleep behavior in humans through long periods of experiment, animal laboratory studies were ultimately critical. At present, however, while researches about the relationship between CSD and glucose homeostasis were mainly clinical studies; animal laboratory studies in the related field were scarce [17]. In addition, the duration span of CSD on animals in previous study, which was much shorter than 1 month [17], does not meet the condition well that people with CSD were suffering from. Thus, in this study, we aimed to further investigate the effects of long-term CSD on glucose homeostasis in rats. The modified multiple platform method (MMPM) was selected to induce CSD. After 3-month intervention, the intraperitoneal glucose tolerance test (IPGTT), insulin tolerance test (ITT), and homeostasis model assessment-IR (HOMA-IR) were performed to evaluate the glucose tolerance and insulin sensitivity.

## Materials and methods

### Experimental design

Twenty-four female 5-month-old Sprague–Dawley rats (weighing 286–324 g) were purchased from the Laboratory Animal Center of Military Medical Science Academy of the PLA (Beijing, China). The animals were maintained on a 12-h light/dark cycle with a controlled temperature (22–24 °C) and housed in 6 cages with 4 rats each. Food and water were available except for 12-h fasting period before the intraperitoneal glucose tolerance test (IPGTT), insulin tolerance test (ITT), and blood samples collection. Body weight was measured weekly. This study was carried out in strict accordance with the recommendations in the Guide for the Care and Use of Laboratory Animals of the National Institutes of Health. The animal use protocol has been reviewed and approved by the Institutional Animal Care and Use Committee (IACUC) of the 309 Hospital of PLA.

The animals were randomly divided into CSD group and control (CON) group (*n* = 12 per group). All animals were adapted to the laboratory conditions for 1 week; in addition, the CSD group rats were acclimated to the sleep deprivation equipment for 30 min per day before the start of the experiment. Then, the CSD rats were placed on multiple small platforms during the procedure, while the CON rats were placed on a grid under the same conditions.

IPGTT and ITT were performed after 3 month of chronic sleep deprivation. Then, the rats were decapitated after a 12-h fasting period. The blood samples was collected between 8:00 and 10:00 o’clock, centrifuged at 1500 *g* for 10 min at 4 °C, and the serum was stored immediately at −80 °C for biochemical parameters tests described below.

### CSD treatment

The CSD rats were intervened by the modified multiple platform method (MMPM), which was proved available to build the CSD model [26]. The animals were group housed (six rats in each arena) in modified multiple platform arenas during CSD. The water tank (123 × 44 × 44 cm^3^) was made of organic glass, containing 12 narrow circular platforms (6.5 cm in diameter), with water up to 1 cm of their upper surface. Thus, the rats could move around freely inside the tank by jumping from one platform to another. When they reached the paradoxical phase of sleep, their faces would touch the water because of the muscle atonia, and kept them awakened. Thus, the paradoxical sleep of rats was deprived to build the CSD model. For the CON group, the housing condition was similar, but there was a grid floor covering on the narrow circular platforms. The grid, which was made of stainless steel with the rods set 2 cm apart, established a different environment where the rats could lie down without falling into the water, albeit their tails may touch the water. After 1 week of adaptation, the rats were placed in the MMPM for 18 h (starting at 16:00 o’clock) every day. After each 18-h sleep deprivation period, the animals were taken back to their individual home cages and allowed to sleep for 6 h (beginning at 10:00 o’clock).

During 3-month environment period, the water tank room was also managed on a 12-h light/dark cycle (lights on at 7:00 h and off at 19:00 h) with a controlled temperature (22–24 °C), and the water in the tank was changed daily during the experiment period. When rats of both two groups were housed in modified multiple platform arenas, chow pellets and water bottles were located at the top of the tank to provide food and water freely.

### IPGTT

To evaluate glucose tolerance, IPGTT was performed after 3-month intervention [27]. The rats were given intraperitoneal glucose (2 g/kg) after a 12-h fasting period. Blood samples, taken from the tip of the tail at 0, 30, 60, 90, and 120 min after glucose injection, were then tested with a portable glucose monitor (Johnson Co. Ltd., China). The receiver operating characteristic (ROC) curves for IPGTT were established to show the variation tendency, and the areas under the curves (AUC) were calculated to evaluate the differences of glucose tolerance between the two groups.

### ITT

After the IPGTT, the rats were again fasted for 12 h before the intraperitoneal insulin (Insulin aspart, Novo Nordisk, China) injection at the dose of 0.75 IU/kg. Tail blood samples were collected as described in IPGTT section and tested at 0, 15, 30, 60, and 90 min after the intraperitoneal injection of insulin. The ROC curves for ITT were also established to show the variation tendency, and the AUC were calculated to evaluate the differences of insulin sensitivity between the two groups.

### Biochemical parameters and homeostasis model assessment-IR (HOMA-IR)

The serum was obtained and stored at −80 °C as described in previous section. Glucose, aspartate aminotransferase (AST), alanine aminotransferase (ALT), creatinine, triglyceride (TG), total cholesterol (TC), LDL-cholesterol, and HDL-cholesterol were determined by using a Beckman Coulter AU5800 Clinical Chemistry analyzer (USA). Fasting serum insulin was measured using an ELISA kit (Beijing Bioco Laibo Technology Co., Ltd, China). The homeostasis model assessment for insulin resistance (HOMA-IR), which was an indication of basal insulin sensitivity, was calculated as published previously (HOMA-IR = [fasting glucose (mmol/L) × fasting insulin (μU/mL)]/22.5) [28].

### Statistical analysis

Data were presented as mean ± SD, and the differences between the CSD group and CON groups were analyzed by the independent sample t-tests. Nonparametric data, such as HOMA-IR, were analyzed by the Mann–Whitney rank sum test. All analyses were performed in SPSS (version 17.0). Statistical significance was set as *P* < 0.05.

## Results

### Body weight

**Fig. 1:**
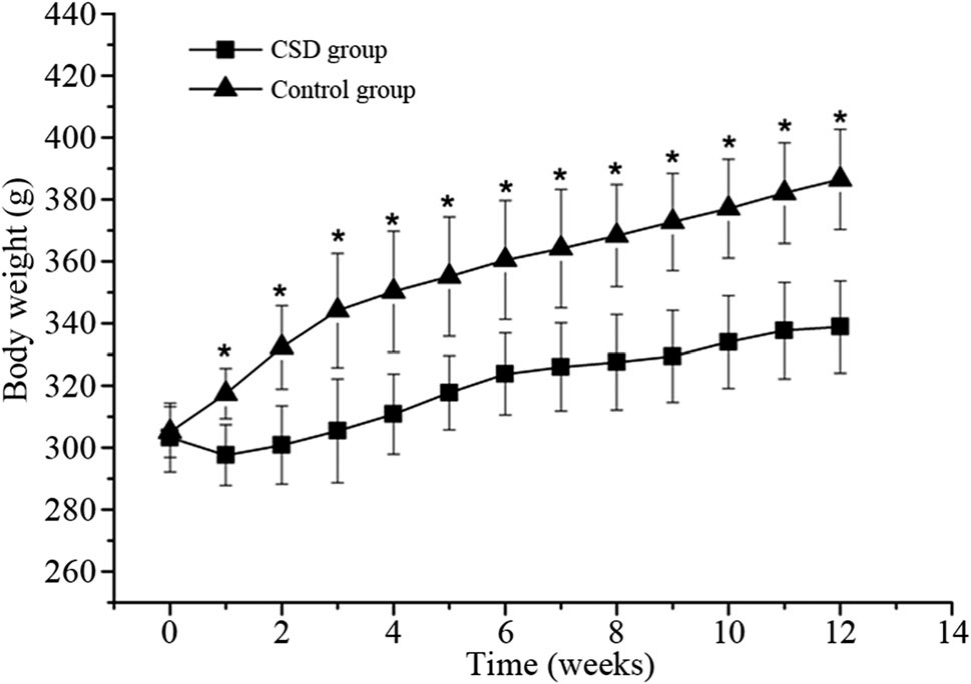
ROC curves for body weights of the two groups. *P < 0.01 compared with the control group

Body weights, checked weekly throughout the 3-month experiment period, were shown in Fig. 1. The CSD animals were significantly lower in body weight than the CON animals already after 1 week of sleep deprivation (*P* < 0.01).

### Biochemical parameters

Table 1 shows the values of serum ALT, AST, CRE, TG, TC, LDL-cholesterol, and HDL-cholesterol. There were no significant difference of biochemical parameters between the CSD and CON groups (*P* > 0.05), except for TG (*P* < 0.01). The fasting plasma glucose of CSD group was higher than that of CON group (*P* < 0.01) (Table 1).

**Table 1:** Biochemical parameters and HOMA-IR of the rats in two groups

Groups	ALT (U/L)	AST (U/L)	CRE (uM)	TG (mM)	TC (mM)	LDL-C (mM)	HDL-C (mM)	Glucose	HOMA-IR
CSD	62.38 ± 9.33	139.14 ± 12.80	34.91 ± 2.73	0.32 ± 0.07*	2.18 ± 0.30	0.66 ± 0.08	1.41 ± 0.19	6.53 ± 0.55*	3.04 ± 0.53*
Control	64.10 ± 9.89	135.73 ± 16.55	33.88 ± 3.31	0.49 ± 0.15	2.25 ± 0.32	0.68 ± 0.09	1.47 ± 0.21	5.14 ± 0.54	2.46 ± 0.36

Data were presented as mean ± SD

*CSD* chronic sleep deprivation, *ALT* alanine aminotransferase, *AST* aspartate aminotransferase, *CRE* creatinine, *TG* triglyceride, *TC* total cholesterol, *LDL-C* LDL-cholesterol, *HDL-C* HDL-cholesterol, *HOMA-IR* homeostasis model assessment-insulin resistance index

* *P* < 0.01 compared with the control group

### IPGTT

**Fig. 2:**
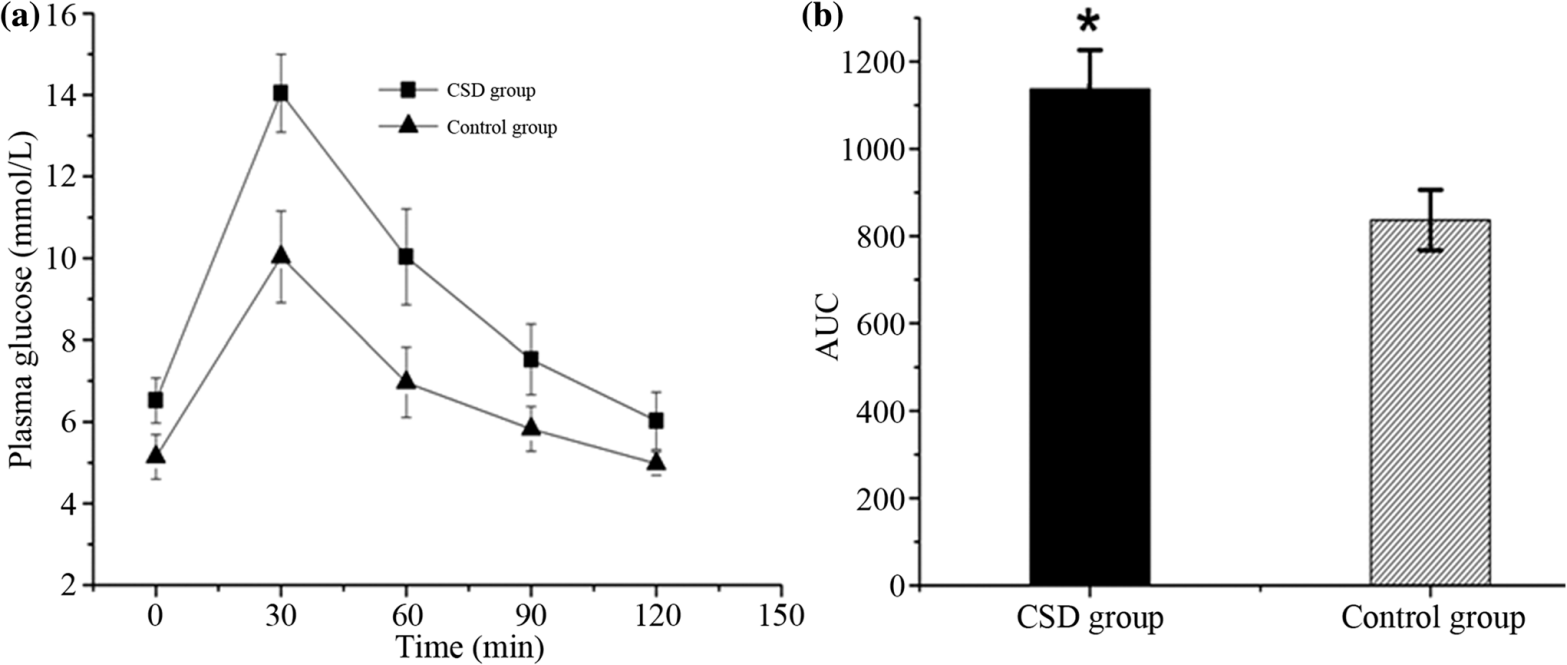
ROC curves for IPGTT of the two groups (A). AUC of ROC curves for IPGTT (B). *P < 0.01 compared with the control group

The ROC curves for IPGTT, established with the Origin 9.0 software, show that the plasma glucose of CSD group was higher than that of CON group (Fig. 2a), and the AUC of ROC curves for IPGTT between the CSD and CON group was significantly different (Fig. 2b, *P* < 0.01).

### Changes in insulin sensitivity

**Fig. 3:**
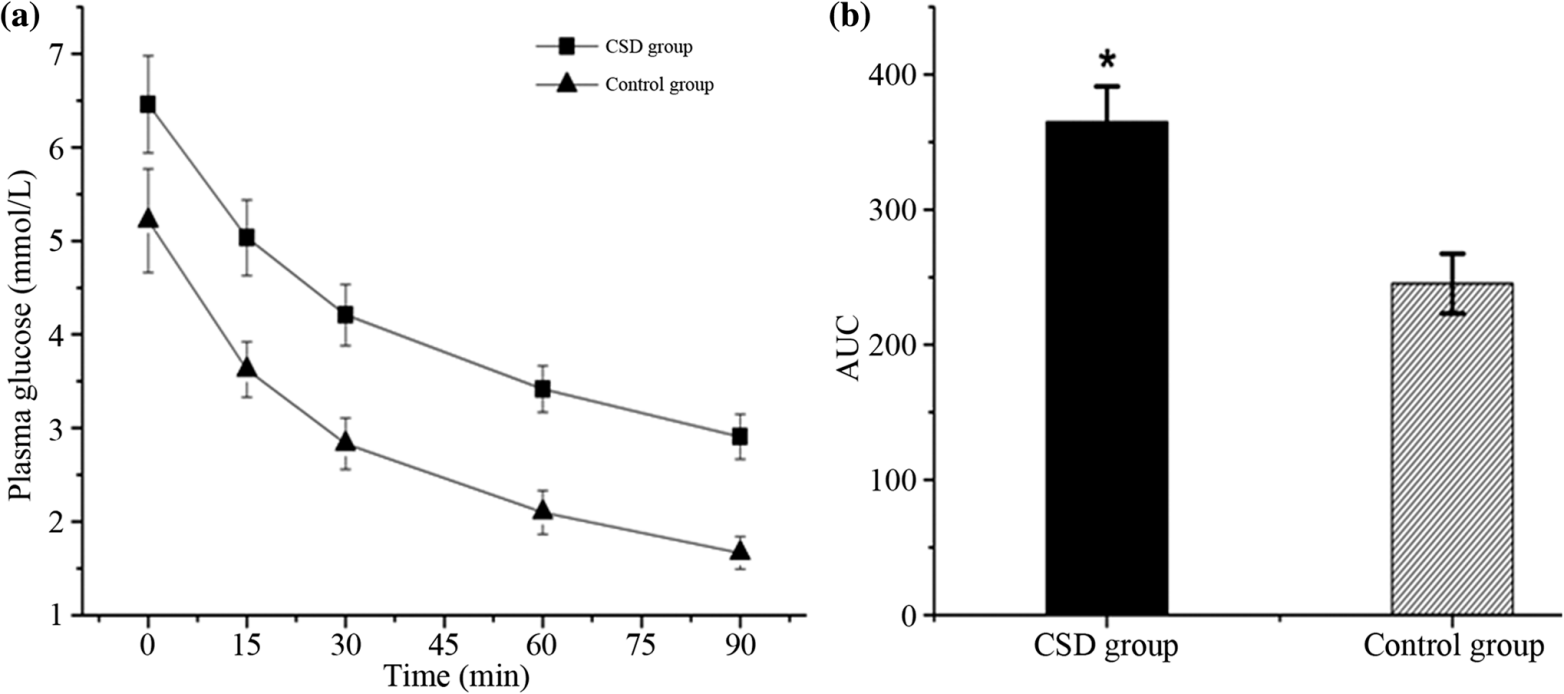
ROC curves for ITT of the two groups (**a**). AUC of ROC curves for ITT (**b**). *P < 0.01 compared with the control group

In the present study, the HOMA-IR and ITT were selected to evaluate the changes in insulin sensitivity. The CSD rats showed an increase in HOMA-IR, and the difference between the two groups was significant (Table 1). The ROC curves for ITT show that the blood glucose of CSD group was higher than that of CON group, and the blood glucose level of CON group fell faster than that of CSD group (Fig. 3a). The AUC of ROC curves for ITT between the CSD and CON group was significantly different (Fig. 3b, *P* < 0.01).

## Discussion

These present results demonstrate that the CSD, leading to both short sleep duration and poor sleep quality, has an important effect on glucose homeostasis in rats. The CSD resulted in an attenuation of weight gain, which may be caused by an increase in energy expenditure [13]. The glucose intolerance, which was the first step in the development of type 2 diabetes, was observed in CSD rats with the IPGTT after 3-month intervention. Furthermore, the CSD rats showed a marked increase in HOMA-IR and ITT, which demonstrated that the rats had decreased their insulin sensitivity.

The modified multiple platform method (MMPM) and rotating device were the most widely used two kinds of methods for chronic sleep disturbances in previous studies. In this study, we used the MMPM for CSD. The MMPM, causing paradoxical sleep deprivation to rats without leading to any additional stress, such as social isolation or instability [29], could induce psychological stress and activate the hypothalamic–pituitary–adrenal (HPA) axis [30]. In addition, the span of this study, lasting 3 months, was longer than most other animal experiment studies about the relationship between CSD and glucose homeostasis [15, 17, 31, 32]. Therefore, this animal model of CSD was similar to the enduring situation that people with chronic sleep disturbances were suffering from, making the data more reliable.

Several clinic-based studies on the association between chronic sleep disturbances and glucose homeostasis have shown that both short sleep duration and poor sleep quality may result in glucose intolerance [33–35]. In our study, the ROC curves for IPGTT showed that the plasma glucose of CSD group was higher than that of CON group, indicating an impaired glucose tolerance. The result was consistent with some previous animal experiment studies [17, 32]. The relationship between sleep duration and insulin sensitivity was uncertain. Some studies have shown that short sleep duration was independently associated with increased insulin resistance, [36–38] while some other studies revealed that long but not short sleep duration is associated with insulin resistance [39, 40]. In addition, according to a recent research, sleep duration was shown to have a U-shaped relationship with insulin resistance [41]. However, in the present study, the CSD rats showed an increase in HOMA-IR and ITT, which indicated that CSD was linked to insulin resistance. In addition, the difference of most serum lipid parameters between two groups was not significant, while the CSD animals were lower in body weight than the CON group already after first week of sleep deprivation. This finding was contrary to some clinic researches, but similar to the preceding animal experiment studies [13, 19].

To date, there is no clear mechanism to explain the association between CSD and glucose homeostasis. The CSD could induce psychological stress which may impair glucose tolerance and associated with the reduction of insulin secretion [42]. The association between psychological stress and glucose homeostasis may involve fibroblast growth factor receptors (FGFRs) [42]. The CSD could also activate the hypothalamic–pituitary–adrenal (HPA) axis which may affect the levels of plasma cortisol (or corticosterone in animal). Some studies showed that plasma cortisol (or corticosterone in animal) of CSD was elevated together with circulating catecholamines [30, 43–45]. In addition, it was reported that raised cortisol (or corticosterone in animal) was associated with glucose tolerance [45–48]. However, some studies reported impaired glucose homeostasis in sleep restricted individuals without changing of cortisol levels [49, 50]. It needs further studies to verify the relationship. Glucose homeostasis is regulated primarily by insulin and insulin sensitivity. Although the reduced insulin response might be a reason for the elevated glucose levels, it has been reported that the rats with glucose intolerance had no changes in plasma insulin response after sleep disturbance [17], making this explanation less likely. In this study, insulin resistance was observed in CSD rats, which could interpret the elevated glucose levels. It was proved in several clinic researches that chronic sleep disturbance are related to obesity and hyperlipemia, which may reduce the insulin sensitivity [19]. However, according to this research and other animal experiment studies, the chronic sleep disturbance resulted in an attenuation of weight gain without hyperlipemia, it implied that the body weight and serum lipid cannot explain the insulin resistance after chronic sleep disturbance [17, 31]. When the body weight gain of CSD rats reduced, the food intake of CSD rats had no changes or increased, suggesting that CSD leads to increased energy expenditure [17, 51], whereas, it was reported that elevated energy expenditure could improve glucose control but not increase the insulin resistance [52]. A few potential pathways have been suggested to contribute to insulin resistance after chronic sleep disturbance, including increased sympathetic activity, elevated evening cortisol levels, increased growth hormone secretion, and an altered inflammatory state [38, 53]. The possible mechanisms may involve reactive oxygen species (ROS), Interleukin-6 (IL-6), tumor necrosis factor-α (TNF-α), and β-cell dysfunction, et al. [54]. Nonetheless, the causal factors explaining the relation between chronic sleep disturbance and insulin resistance need an additional study to certify, and most likely include central autonomic pathways.

Some limitations of the present study deserve attention. First, although the insulin response might refer to the mechanism of the glucose intolerance, we did not evaluate the insulin levels after IPGTT. We should pay attention to examine this question in future studies. Second, we did not study the glucose homeostasis after dozens of days or several months; thus, it was unclear that whether the glucose intolerance and insulin resistance would improve after the recovery of sleep. Future studies should verify that it is a reversible process or not. Third, we did not collect the data of food intake and brain temperature. It was reported that the food intake of CSD rats had no changes or increased and the brain temperature remained at a higher level, whereas the body weight reduced, which may suggest that CSD leads to increased energy expenditure [17, 32, 52]. However, it was discussed in previous part that the increased energy expenditure was not the reason of glucose intolerance and insulin resistance. Fourth, we did not evaluate the effect of acute sleep deprivation on glucose homeostasis; therefore, it was difficult to determine whether the changes in glucose homeostasis were unique to the influence of chronic total sleep deprivation or were reflective of paradoxical sleep deprivation. However, it was reported in previous study that the acute sleep disturbance (24 h) could lead to hyperglycemia without changes in the insulin response [17]. Finally, the sample size is relatively small, and the results should be considered with caution.

In conclusion, our data reveal that the CSD, leading to both short sleep duration and poor sleep quality, has a marked effect on glucose homeostasis, comprising of glucose intolerance and insulin resistance. We should avoid a disturbance of the normal sleep pattern to prevent the disorder of glucose homeostasis. Meanwhile, further study of the relation of CSD and type 2 diabetes should also be given high priority in the future.

## References

[1] Broman JE, Lundh LG, Hetta J (1996). Insufficient sleep in the general population. Neurophysiol Clin.

[2] Groeger JA, Zijlstra FR, Dijk DJ (2004). Sleep quantity, sleep difficulties and their perceived consequences in a representative sample of some 2000 British adults. J Sleep Res.

[3] Bonnet MH, Arand DL (1995). We are chronically sleep deprived. Sleep.

[4] Worthman CM, Brown RA (2013). Sleep budgets in a globalizing world: biocultural interactions influence sleep sufficiency among Egyptian families. Soc Sci Med.

[5] Ju SY, Choi WS (2013). Sleep duration and metabolic syndrome in adult populations: a meta-analysis of observational studies. Nutr Diabetes.

[6] Cappuccio FP, D’Elia L, Strazzullo P, Miller MA (2010). Sleep duration and all-cause mortality: a systematic review and meta-analysis of prospective studies. Sleep.

[7] Rod NH, Kumari M, Lange T, Kivimäki M, Shipley M, Ferrie J (2014). The joint effect of sleep duration and disturbed sleep on cause-specific mortality: results from the Whitehall II cohort study. PLoS One.

[8] McEwen BS (2006). Sleep deprivation as a neurobiologic and physiologic stressor: allostasis and allostatic load. Metabolism.

[9] Okun ML, Coussons-Read M, Hall M (2009). Disturbed sleep is associated with increased C-reactive protein in young women. Brain Behav Immun.

[10] Irwin MR (2015). Why sleep is important for health: a psychoneuroimmunology perspective. Annu Rev Psychol.

[11] Bernert RA, Kim JS, Iwata NG, Perlis ML (2015). Sleep disturbances as an evidence-based suicide risk factor. Curr Psychiatry Rep.

[12] Nedeltcheva AV, Kilkus JM, Imperial J, Kasza K, Schoeller DA, Penev PD (2009). Sleep curtailment is accompanied by increased intake of calories from snacks. Am J Clin Nutr.

[13] Barf RP, Van Dijk G, Scheurink AJ, Hoffmann K, Novati A, Hulshof HJ, Fuchs E, Meerlo P (2012). Metabolic consequences of chronic sleep restriction in rats: changes in body weight regulation and energy expenditure. Physiol Behav.

[14] Troxel WM, Buysse DJ, Matthews KA, Kip KE, Strollo PJ, Hall M, Drumheller O, Reis SE (2010). Sleep symptoms predict the development of the metabolic syndrome. Sleep.

[15] Vetrivelan R, Fuller PM, Yokota S, Lu J, Saper CB (2012). Metabolic effects of chronic sleep restriction in rats. Sleep.

[16] Dominguez-Rodriguez A, Abreu-Gonzalez P (2014). The link between sleep duration and inflammation: effects on cardiovascular disease. Int J Cardiol.

[17] Barf RP, Meerlo P, Scheurink AJ (2010). Chronic sleep disturbance impairs glucose homeostasis in rats. Int J Endocrinol.

[18] Dash MB, Bellesi M, Tononi G, Cirelli C (2013). Sleep/wake dependent changes in cortical glucose concentrations. J Neurochem.

[19] Liu J, Hay J, Faught BE (2013). The association of sleep disorder, obesity status, and diabetes mellitus among US adults—the NHANES 2009–2010 survey results. Int J Endocrinol.

[20] Matthews KA, Dahl RE, Owens JF, Lee L, Hall M (2012). Sleep duration and insulin resistance in healthy black and white adolescents. Sleep.

[21] Gonzalez-Ortiz M, Martinez-Abundis E, Balcazar-Munoz BR, Pascoe-Gonzalez S (2000). Effect of sleep deprivation on insulin sensitivity and cortisol concentration in healthy subjects. Diabetes Nutr Metab.

[22] Punjabi NM, Shahar E, Redline S, Gottlieb DJ, Givelber R, Resnick HE (2004). Sleep-disordered breathing, glucose intolerance, and insulin resistance: the Sleep Heart Health Study. Am J Epidemiol.

[23] Kim CR, Song YM, Shin JY, Gim W (2016). Association between sleep duration and impaired fasting glucose in korean adults: results from the korean national health and nutrition examination survey 2011–2012. Korean J Fam Med.

[24] Baoying H, Hongjie C, Changsheng Q, Peijian W, Qingfei L, Yinghua L, Huibin H, Jixing L, Liantao L, Ling C, Kaka T, Zichun C, Lixiang L, Jieli L, Yufang B, Guang N, Penli Z, Junping W, Gang C (2014). Association of napping and night-time sleep with impaired glucose regulation, insulin resistance and glycated haemoglobin in Chinese middle-aged adults with no diabetes: a cross-sectional study. BMJ Open.

[25] Lou P, Chen P, Zhang L, Zhang P, Chang G, Zhang N, Li T, Qiao C (2014). Interaction of sleep quality and sleep duration on impaired fasting glucose: a population-based cross-sectional survey in China. BMJ Open.

[26] Oh MM, Kim JW, Jin MH, Kim JJ, du Moon G (2012). Influence of paradoxical sleep deprivation and sleep recovery on testosterone level in rats of different ages. Asian J Androl.

[27] Kim SJ, Ju A, Lim SG, Kim DJ (2013). Chronic alcohol consumption, type 2 diabetes mellitus, insulin-like growth factor-I (IGF-I), and growth hormone (GH) in ethanol-treated diabetic rats. Life Sci.

[28] Cacho J, Sevillano J, de Castro J, Herrera E, Ramos MP (2008). Validation of simple indexes to assess insulin sensitivity during pregnancy in Wistar and Sprague–Dawley rats. Am J Physiol Endocrinol Metab.

[29] Machado RB, Suchecki D, Tufik S (2006). Comparison of the sleep pattern throughout a protocol of chronic sleep restriction induced by two methods of paradoxical sleep deprivation. Brain Res Bull.

[30] Ma C, Wu G, Wang Z, Wang P, Wu L, Zhu G, Zhao H (2014). Effects of chronic sleep deprivation on the extracellular signal-regulated kinase pathway in the temporomandibular joint of rats. PLoS One.

[31] Alvarenga TA, Tufik S, Pires GN, Andersen ML (2012) Influence of food restriction on lipid profile and spontaneous glucose levels in male rats subjected to paradoxical sleep deprivation. Clinics (Sao Paulo, Brazil) 67: 375–80. 10.6061/clinics/2012(04)11 PMC3317256 22522763

[32] Baud MO, Magistretti PJ, Petit JM (2013). Sustained sleep fragmentation affects brain temperature, food intake and glucose tolerance in mice. J Sleep Res.

[33] Tanno S, Tanigawa T, Saito I, Nishida W, Maruyama K, Eguchi E, Sakurai S, Osawa H, Punjabi NM (2014). Sleep-related intermittent hypoxemia and glucose intolerance: a community-based study. Sleep Med.

[34] Qiu C, Enquobahrie D, Frederick IO, Abetew D, Williams MA (2010). Glucose intolerance and gestational diabetes risk in relation to sleep duration and snoring during pregnancy: a pilot study. BMC Women’s Health.

[35] Lai YJ, Lin CL, Lin MC, Lee ST, Sung FC, Chang YJ, Kao CH (2013). Population-based cohort study on the increase in the risk for type 2 diabetes mellitus development from nonapnea sleep disorders. Sleep Med.

[36] Zuo H, Shi Z, Yuan B, Dai Y, Hu G, Wu G, Hussain A (2012). Interaction between physical activity and sleep duration in relation to insulin resistance among non-diabetic Chinese adults. BMC Public Health.

[37] Liu R, Zee PC, Chervin RD, Arguelles LM, Birne J, Zhang S, Christoffel KK, Brickman WJ, Zimmerman D, Wang B, Wang G, Xu X, Wang X (2011). Short sleep duration is associated with insulin resistance independent of adiposity in Chinese adult twins. Sleep Med.

[38] Spiegel K, Knutson K, Leproult R, Tasali E, Van Cauter E (2005). Sleep loss: a novel risk factor for insulin resistance and Type 2 diabetes. J Appl Physiol (1985).

[39] Chang JK, Koo M, Kao VY, Chiang JK (2012). Association of sleep duration and insulin resistance in Taiwanese vegetarians. BMC Public Health.

[40] Pyykkönen AJ, Isomaa B, Pesonen AK, Eriksson JG, Groop L, Tuomi T, Räikkönen K (2014). Sleep duration and insulin resistance in individuals without type 2 diabetes: the PPP-Botnia study. Ann Med.

[41] Ohkuma T, Fujii H, Iwase M, Ogata-Kaizu S, Ide H, Kikuchi Y, Idewaki Y, Jodai T, Hirakawa Y, Nakamura U, Kitazono T (2014). U-shaped association of sleep duration with metabolic syndrome and insulin resistance in patients with type 2 diabetes: the Fukuoka Diabetes Registry. Metabolism.

[42] Rojas JM, Matsen ME, Mundinger TO, Morton GJ, Stefanovski D, Bergman RN, Kaiyala KJ, Taborsky GJ, Schwartz MW (2015). Glucose intolerance induced by blockade of central FGF receptors is linked to an acute stress response. Mol Metab.

[43] Spiegel K, Leproult R, L’hermite-Balériaux M, Copinschi G, Penev PD, Van Cauter E (2004). Leptin levels are dependent on sleep duration: relationships with sympathovagal balance, carbohydrate regulation, cortisol, and thyrotropin. J Clin Endocrinol Metab.

[44] Buxton OM, Cain SW, O’Connor SP, Porter JH, Duffy JF, Wang W, Czeisler CA, Shea SA (2012). Adverse metabolic consequences in humans of prolonged sleep restriction combined with circadian disruption. Sci Transl Med.

[45] Nedeltcheva AV, Kessler L, Imperial J, Penev PD (2009). Exposure to recurrent sleep restriction in the setting of high caloric intake and physical inactivity results in increased insulin resistance and reduced glucose tolerance. J Clin Endocrinol Metab.

[46] Hackett RA, Kivimäki M, Kumari M, Steptoe A (2016). Diurnal cortisol patterns, future diabetes, and impaired glucose metabolism in the Whitehall II cohort study. J Clin Endocrinol Metab.

[47] Risberg A, Sjöquist M, Wedenberg K, Larsson A (2016). Elevated glucose levels in early puerperium, and association with high cortisol levels during parturition. Scand J Clin Lab Invest.

[48] Zhao JP, Lin H, Jiao HC, Song ZG (2009). Corticosterone suppresses insulin- and NO-stimulated muscle glucose uptake in broiler chickens (*Gallus gallus domesticus*). Comp Biochem Physiol C: Toxicol Pharmacol.

[49] Buxton OM, Pavlova M, Reid EW, Wang W, Simonson DC, Adler GK (2010). Sleep restriction for 1 week reduces insulin sensitivity in healthy men. Diabetes.

[50] Donga E, van Dijk M, van Dijk JG, Biermasz NR, Lammers GJ, van Kralingen KW, Corssmit EP, Romijn JA (2010). A single night of partial sleep deprivation induces insulin resistance in multiple metabolic pathways in healthy subjects. J Clin Endocrinol Metab.

[51] Everson CA, Folley AE, Toth JM (2012). Chronically inadequate sleep results in abnormal bone formation and abnormal bone marrow in rats. Exp Biol Med (Maywood).

[52] Bjursell M, Wedin M, Admyre T, Hermansson M, Böttcher G, Göransson M, Lindén D, Bamberg K, Oscarsson J, Bohlooly YM (2013). Ageing Fxr deficient mice develop increased energy expenditure, improved glucose control and liver damage resembling NASH. PLoS One.

[53] Donga E, Romijn JA (2014). Sleep characteristics and insulin sensitivity in humans. Handb Clin Neurol.

[54] Briançon-Marjollet A, Weiszenstein M, Henri M, Thomas A, Godin-Ribuot D, Polak J (2015). The impact of sleep disorders on glucose metabolism: endocrine and molecular mechanisms. Diabetol Metab Syndr.

